# Supplementary material for: Complexome profiling of the Chlamydomonas psb28 mutant reveals TEF5 as an early PSII assembly factor
Source: Plant Cell. 2025 Mar 17;37(6):koaf055. doi: 10.1093/plcell/koaf055 (PMC12164590; doi:10.1093/plcell/koaf055)
Supplement: koaf055_Supplementary_Data [file koaf055_supplementary_data.zip › psb28_interactive.zip!/plots/intensity/qualityPlots/peptides/nad1_Cre-2.g801499.t1.p1.html]

### Cre-2.g801499.t1.p1

Trivial name: nad1  
SeqCoverage [median]: 5.50  
Mass: 31.59 kDa  
  
 

| Sequence | MissedCleavage | Mass [Da] | wtIntensity | muIntensity | Score |
| --- | --- | --- | --- | --- | --- |
| TVMASMQR | 0 | 922.44 | 20241.50 | 9801.86 | 117.40 |
| YRYDQFMR | 1 | 1177.53 | 7330.54 | 7488.64 | 93.43 |
|  |  |  | 27572.0 | 17290.5 |  |
